# Safe prescribing of non-steroidal anti-inflammatory drugs in patients with osteoarthritis – an expert consensus addressing benefits as well as gastrointestinal and cardiovascular risks

**DOI:** 10.1186/s12916-015-0285-8

**Published:** 2015-03-19

**Authors:** Carmelo Scarpignato, Angel Lanas, Corrado Blandizzi, Willem F Lems, Matthias Hermann, Richard H Hunt, For the International NSAID Consensus Group

**Affiliations:** 1 grid.10383.390000000417580937Department of Clinical & Experimental Medicine, Clinical Pharmacology & Digestive Pathophysiology Unit, University of Parma, Maggiore University Hospital, Cattani Pavillon, I-43125 Parma, Italy; 2 grid.11205.370000000121528769Service of Digestive Diseases, Clinic Hospital Lozano Blesa, Aragón Institute for Health Research (IIS Aragón), CIBERehd, University of Zaragoza, Zaragoza, Spain; 3 grid.5395.a0000000417573729Department of Clinical & Experimental Medicine, Division of Pharmacology & Chemotherapy, University of Pisa, Pisa, Italy; 4 grid.16872.3a000000040435165XDepartment of Rheumatology, VU University Medical Center, Amsterdam, The Netherlands; 5 grid.412004.30000000404789977Department of Cardiology, University Hospital, Zurich, Switzerland; 6 grid.25073.330000000419368227Department of Medicine, Division of Gastroenterology and Farncombe Family Digestive Health Research Institute, McMaster University, Hamilton, ON Canada

**Keywords:** Cardiovascular risk, Consensus, COX-2 selective inhibitors, Gastrointestinal risk, Gastroprotection, Low-dose aspirin, Non-steroidal anti-inflammatory drugs, Osteoarthritis

## Abstract

**Background:**

There are several guidelines addressing the issues around the use of NSAIDs. However, none has specifically addressed the upper versus lower gastrointestinal (GI) risk of COX-2 selective and non-selective compounds nor the interaction at both the GI and cardiovascular (CV) level of either class of drugs with low-dose aspirin. This Consensus paper aims to develop statements and guidance devoted to these specific issues through a review of current evidence by a multidisciplinary group of experts.

**Methods:**

A modified Delphi consensus process was adopted to determine the level of agreement with each statement and to determine the level of agreement with the strength of evidence to be assigned to the statement.

**Results:**

For patients with both low GI and CV risks, any non-selective NSAID (ns-NSAID) alone may be acceptable. For those with low GI and high CV risk, naproxen may be preferred because of its potential lower CV risk compared with other ns-NSAIDs or COX-2 selective inhibitors, but celecoxib at the lowest approved dose (200 mg once daily) may be acceptable. In patients with high GI risk, if CV risk is low, a COX-2 selective inhibitor alone or ns-NSAID with a proton pump inhibitor appears to offer similar protection from upper GI events. However, only celecoxib will reduce mucosal harm throughout the entire GI tract. When both GI and CV risks are high, the optimal strategy is to avoid NSAID therapy, if at all possible.

**Conclusions:**

Time is now ripe for offering patients with osteoarthritis the safest and most cost-effective therapeutic option, thus preventing serious adverse events which could have important quality of life and resource use implications.

Please see related article: http://dx.doi.org/10.1186/s12916-015-0291-x.

**Electronic supplementary material:**

The online version of this article (doi:10.1186/s12916-015-0285-8) contains supplementary material, which is available to authorized users.

## Background

Pain is a common reason for patients to visit their family physician [1,2] and the numbers seeking treatment for pain is anticipated to rise as the population ages and chronic conditions, such as osteoarthritis (OA), increase. In the United Kingdom (UK), annually, more than 17 million prescriptions are written for anti-inflammatory and analgesic drugs [3]. In addition, over-the-counter analgesic (non-steroidal anti-inflammatory drugs (NSAIDs) and paracetamol) use is substantial, with 44% of patients consuming more than the recommended dosage on the label [4]. Musculoskeletal pain is common and disabling, especially in the elderly population, whose number and proportion is estimated to double by 2030, compared to the 2000 figure [5]. Prescribing pain medications within this particular population requires the skill of a knowledgeable physician to navigate through the numerous variables (e.g., physiologic changes, co-morbidities, and co-therapies) that make the elderly a heterogeneous and complex population to treat [6,7].

Over the last few years, professional organizations, including the American College of Rheumatology [8], the American Pain Society [9], and the European League Against Rheumatism [10], have published treatment guidelines to assist clinicians in achieving effective pain management. Safety is a core concern in all these guidelines, especially for chronic conditions, such as OA, that require long-term treatment. Hence, there is a consensus among recommendations that paracetamol (acetaminophen) should be the first-line analgesic agent due to its favorable side effect and safety profile, despite several meta-analyses having shown that it is less effective in pain relief than anti-inflammatory drugs [11-14].

Besides being a less effective analgesic, paracetamol may not be as safe as believed, both from a gastrointestinal (GI) and cardiovascular (CV) perspective, not to mention the well-known hepatotoxicity (especially at doses higher than 3 g daily) [15]. Indeed, a nested case-control study found that use of this compound (any dose) is associated with a small but significant risk of upper GI complications (relative risk [RR], 1.3; 95% confidence interval [CI], 1.1–1.5) [16]. The RR was 3.6 (95% CI, 2.6–5.1) among paracetamol users of more than 2 g daily. In addition, while women from the Nurses’ Health Study, who reported occasional use of paracetamol, did not experience a significant increase in the risk of CV events, those who reported a frequent (6–14 tablets/week) use had a RR of 1.35 (95% CI, 1.14–1.59) [17]. Finally, regular paracetamol use is associated with an increased risk of hypertension both in women [18] and men [19]. More recently, it was also shown that paracetamol, at doses of 3 g daily, induces a significant increase in ambulatory blood pressure in patients with coronary artery disease [20]. The above findings are not surprising in the light of the recent discovery that paracetamol is indeed a selective cyclooxygenase (COX)-2 inhibitor in man [21].

Although OA has long been considered a “wear and tear” disease leading to loss of cartilage, progress in molecular biology in the 1990s has profoundly modified this paradigm. The discovery that many soluble mediators, such as cytokines or prostaglandins, can increase the production of matrix metalloproteinases by chondrocytes, led to the first steps of an “inflammatory” theory [22,23]. Recent experimental data have shown that subchondral bone may have a substantial role in the OA process, as a mechanical dampener, as well as a source of inflammatory mediators implicated both in the OA pain process and in the degradation of the cartilage deep layer [24]. Thus, initially considered cartilage driven, OA, the prototypic age-related disease [25], is much more complex than previously thought and low-grade (local and systemic) inflammation is the hallmark of this chronic and progressing condition [24]. Thus, COX-2 selective or non-selective NSAIDs (ns-NSAIDs), which display both analgesic and anti-inflammatory properties, represent a pathophysiologically sound approach.

NSAIDs are very effective drugs [8-10,26,27], but their use is associated with a broad spectrum of adverse reactions involving the liver, kidney, CV system, skin, and gut [28]. GI side effects are the most common and cover a wide clinical spectrum ranging from dyspepsia, heartburn, and abdominal discomfort to more serious events such as peptic ulcer with life-threatening complications of bleeding and perforation [29,30]. The dilemma for the physician prescribing NSAIDs is, therefore, to maintain the anti-inflammatory and analgesic benefits while reducing or preventing their untoward GI effects.

The use of all medications increases with age and the elderly are at increased risk of adverse drug reactions. The occurrence of GI complications depends on the presence and number of risk factors, and age is the most frequent and relevant of these. Thus, patients at upper GI risk should have preventative strategies in place, including the use of the lowest effective dose of NSAID, co-therapy with a gastroprotective drug, or the use of a COX-2 selective agent [31,32]. However, eradication of associated *Helicobacter pylori* (*H. pylori*) infection has been shown to be beneficial when starting treatment with NSAIDs or aspirin, especially in the presence of an ulcer history [33-35] and was recommended by the latest Maastricht IV/Florence European Consensus report [36]. In contrast, eradication appears not to be useful in patients already under long-term NSAID treatment, which is the most common picture in clinical practice [37]. The best strategy to prevent lower GI complications has yet to be defined.

It is increasingly recognized that the small bowel, like the stomach, is a site for NSAID-associated mucosal lesions, giving rise to NSAID-enteropathy [38]. Over the last 10 years, there has been a decreasing trend in NSAID-induced symptomatic GI events in rheumatic patients [39] and, in line with that, in hospitalizations due to upper GI complications, while lower GI complications showed an apparent increasing trend [40-43]. In addition, the clinical impact and severity of lower GI events have actually been greater than those in the upper GI tract [41]. Recent evidence has also shown that proton pump inhibitors (PPIs) are unable to prevent NSAID-associated intestinal damage and that some COX-2 selective inhibitors display a better intestinal tolerability compared to traditional (i.e. non selective) NSAIDs, with or without acid inhibition [30,44].

During the last few years, great attention has been focused on the adverse CV effects of COX-2 selective NSAIDs, which prompted a re-evaluation of the CV and global safety profile of traditional anti-inflammatory drugs. The increased CV risk of COX-2 selective inhibitors has been well documented in randomized controlled trials (RCTs) and observational studies. While this risk may be different according to dose and patient baseline CV clinical conditions, more recent evidence suggests that at least some, if not all, ns-NSAIDs may also increase that risk [45-48]. The reno-vascular effects of NSAIDs are also well known. Current evidence suggests that ns-NSAIDs and COX-2 selective NSAIDs have a similar incidence of these adverse effects, but with molecule-specific quantitative differences between the various drugs [49].

Although the two processes can be separated at the cellular, tissue, and clinical levels, aging and the development of OA are closely linked [50]. CV risk factors also increase with age [51] and the presence of CV co-morbidity is rather high (up to 44%) in patients with OA [52-54]; out of a large (>17,000 subjects) population of OA patients, 21.4% were low-dose aspirin users [54]. The presence of CV comorbidities and concomitant aspirin use influence NSAID selection among rheumatologists [54,55]; however, NSAID prescribing was not always appropriate or in accordance with current guidelines or recommendations by regulatory agencies [54].

There are several guidelines and expert recommendations addressing the issues around the use of NSAIDs [8,32,56-70]. However, none has specifically addressed the upper versus lower GI risk of COX-2 selective and non-selective compounds or the interaction at both the GI and CV level of either class of drugs with low-dose aspirin. To this end, a multidisciplinary group of experts convened to review current evidence, with the aim of developing statements and guidance devoted to these specific issues, to help clinicians make evidence-based decisions when selecting anti-inflammatory agents for the individual patient.

## Materials and methods

Clinically relevant consensus statements relating to NSAID use were developed according to generally accepted standards [71], following a methodology similar to the one adopted by the Canadian consensus group [68].

In 2011, a series of meetings were held in Amsterdam (the Netherlands) and Treviso (Italy) with a multidisciplinary group of experts, during which key themes around CV and GI risk were identified, current data reviewed, and knowledge gaps recognized. The aim of the meetings was two-fold – i) to identify and address ongoing issues around CV and GI risk associated with NSAIDs, which leave primary care physicians uncertain about how to prescribe these drugs in patients with OA; and ii) to develop practical evidence-based clinical guidance on the appropriate use of NSAID therapy.

Following each meeting, statements to help inform recommendations for the management of pain in patients with OA were drafted. A core group of expert statement leaders (Frank Buttgereit, non-voting Chair of the Consensus Group, Richard H Hunt, Carmelo Scarpignato) gathered supporting evidence and refined the statements following review by and feedback from the wider expert group (Corrado Blandizzi, Ernest Choy, Mart van der Laar, Angel Lanas, Willem F Lems, Andrew Moore). This culminated in the Expert Summit meeting (Amsterdam, November 15–16, 2011), during which 34 experts met, discussed the evidence, and voted on nine clinical statements around the efficacy of NSAIDs and NSAID-associated CV and GI risks. Consensus was reached on all nine statements (i.e., ≥75% agreement, see below).

### Membership of the consensus group

An organizing committee selected a non-voting Chair and an international, multidisciplinary group of 34 voting physicians and evidence-based medicine experts (Additional file 1). Non-voting observers included representatives from the Lucid Group (who provided the logistic support for the meeting as well as editorial assistance) and from the pharmaceutical industry (Additional file 1). Representatives from these groups did not participate in any consensus discussions or in the voting.

### Nature and extent of background preparation

Each statement leader addressed a specific topic and made their own selection of primary references and systematic reviews by searching MEDLINE, EMBASE, and CINAHL databases (from 1990 to October 2011) as well as the proceedings of the major digestive and rheumatology meetings. The writing and references provided by each statement leader were circulated among all the members of the expert group, who sent their written comments prior to the meeting. In reviewing the statements and evidence, experts were encouraged to suggest amendments to the wording of the statements, as well as additions or deletions to the list of references, as they felt appropriate. In either case, they were asked to also state the rational for their suggested amendments.

Once all the feedback was collated, for each statement, a restricted (n = 1 to 3) number of experts (designated as statement group members) provided – during a teleconference – their feedback and suggestions for improvement and/or change of both the wording of each statement and the supporting evidence, taking into account the views of the wider expert group. This revised list of nine statements was brought for discussion at the Consensus meeting.

### Modified Delphi consensus process

The revised (preliminary) statements were circulated electronically for review before the meeting. A summary of the evidence was presented by each statement leader during the meeting [72].

At the end of each presentation, an in-depth discussion, driven by the Chair, was held with two objectives, namely i) to clarify the meaning of the statement or to reduce any ambiguity and ii) to clarify any queries regarding the evidence. On some occasions this resulted in further modification of the statement. The final statement was then voted on in two parts. The first vote was to determine the level of agreement with the statement itself and the second to determine the level of agreement with the strength of evidence to be assigned to the statement. All voting at the meeting was conducted using keypads to ensure that the process was anonymous. Experts were asked to indicate a level of agreement with the statement on a scale of ‘a’ to ‘f’, ranging from strongly agree to strongly disagree. Statements were accepted when more than 75% of participants voted ‘a’, ‘b’, or ‘c’ (agree strongly, agree moderately, or agree mildly). The strength of evidence was assessed as suggested by the GRADE working group (Table 1) and each statement was assigned a grade to indicate the quality of evidence [73]. Evidence grades were recorded as voted for by the experts.

**Table 1:** **Grading of the quality of the evidence based on the GRADE system**

**Nature of evidence**	**Study design**	**Study execution**	**Consistency**	**Directness of evidence**
A	Pairwise meta-analysis of comparative randomized controlled trials (RCTs) (for interventions)	No important flaws	Consistent	Direct or strong indirect
	RCTs (for interventions)			
	Non-randomized studies (for diagnosis and prognosis)			
B	Meta-analysis of RCTs or RCTs (for interventions)	Important flaw < OR > Inconsistent < OR > Weak indirect		
	Non-randomized studies (for diagnosis or prognosis)	Important flaw < OR > Inconsistent < OR > Weak indirect		
	Non-randomized controlled studies (for interventions)	No important flaws consistent direct < OR > Strong indirect		
C	Non-randomized controlled studies (for interventions)	Important flaw < OR > Inconsistent < OR > Weak indirect		
	Meta-analyses or RCTs with a combination of important flaws AND inconsistency AND/OR indirect evidence			
D	Other evidence (not expert opinion)			
E	Expert opinion			
**Exceptions that can alter the quality of grading**				
Sparse data (few events); use of data not in its initial randomization or apparent publication bias can lower the quality; a very strong association can raise the quality				
**Coding notes**				
Important flaws occur when the highest standards of research that could be achieved by a study are not applied				
Consistency occurs at two levels – design: consistent methods, patients, outcomes; and statistical: a test of homogeneity of a summary estimate when the level of design consistency is acceptable and meta-analysis appropriate				
Directness – direct evidence: relevant patient benefits and harms are measured in studies; strong indirect: the surrogate endpoint is strongly related to desirable endpoints, or direct evidence is available for a sufficiently related patient group; weak indirect: the relationship between the study outcomes and patient benefits or harms is insufficient				
**Summary of quality of evidence**				
A. High quality of evidence – future evidence is unlikely to change confidence in the estimate of effect				
B. Moderate quality of evidence – future evidence is likely to have an impact on the confidence of the estimate of effect and may change that estimate				
C. Poor quality evidence – future evidence is very likely to have an impact on the confidence of the estimate of effect and is likely to change that estimate				
D. and E. Very poor quality evidence – Any estimate of effect is very uncertain				

Developed from Lomas J, 1991 [71].

### General meeting organization

A 2-day consensus conference was held on November 15 to 16, 2011, in accordance with generally accepted standards for the development of clinical practice guidelines [74]. Statements of conflicts of interest were obtained from all voting participants [75].

Lucid Group (London, UK) managed all the organization, the audio recording and the electronic voting system. The Consensus conference and the previous expert meetings were supported through an unrestricted educational grant from Pfizer Europe, who entrusted Lucid with the management of the whole process.

### Preparation of the consensus paper

A core working group of experts drafted the manuscript (CS, AL, CB, RH), which was then reviewed by all the statements leaders and the authors, who all approved the final draft.

## Results

### Presentation of the statements

Each statement is followed by the ‘grade’ of supporting evidence and the results of the two votes: i) agreement with the statement and ii) agreement with the grade of evidence followed by a brief summary. A consensus was considered reached when more than 75% of participants voted a, b, or c (agree strongly, agree moderately, or agree mildly).

### NSAIDs: efficacy in OA and impact on quality of life (QoL)

**Statement 1:***OA impacts quality and quantity of life; it should therefore be treated appropriately.***Level of Agreement:** Strong Agreement (vote statement: a, 48.5%; b, 36.4%; c, 12.1%; d, 3%). **Level of Evidence:** B (vote grade: A, 42.4%; B, 48.5%; C, 9.1%).

Pain strongly and negatively impacts QoL, with increasing evidence that it may also impact quantity of life. Chronic pain interferes with the domains of physical and social functioning, emotional and mental health, energy, vitality, and general health [76]. In addition, it has been shown to be indirectly associated with a reduced cumulative survival due to CV and respiratory causes [77].

QoL is adversely impacted by OA and this has been shown in large, comprehensive, cross-sectional, or longitudinal studies [78], and this is supported by many other studies confirming the same conclusions. OA patients suffer a spectrum of symptoms, which impair normal daily functions including sleeping, walking, climbing stairs, opening containers, preparing food, or self-caring to variable extents [78]. There is evidence that chronic musculoskeletal disorders, including OA, have an overall greater adverse impact on QoL than other chronic diseases such as CV, respiratory, cerebrovascular, and neurologic disorders, GI diseases, and cancer [79].

Three large, comprehensive, longitudinal studies confirm the effect of OA on longevity [77,80-82]. Reduced physical function, especially walking disability, is a major risk factor for mortality in OA patients [81]. Thus, physical activity is good for health [80]. The potential impact of disability on longevity has also been demonstrated in other musculoskeletal inflammatory conditions. In a cohort of patients with ankylosing spondylitis followed for up to 33 years, the highest mortality was associated with greater disability and with non-use of NSAIDs [83].

Large observational studies and analyses of RCTs or pooled data confirm the important benefits of treating OA [84-86]. Treatment of OA improves physical performance and is beneficial to several domains of arthritis function, including pain, stiffness, function, and global ratings of performance. In addition, it improves the quality of sleep [87-89].

**Statement 2:***Many OA patients requiring NSAID therapy are not being treated appropriately according to their GI risk profile.***Level of Agreement:** Strong Agreement (vote statement: a, 75.0%; b, 21.9%; c, 3.1%). **Level of Evidence:** A (vote grade: A, 68.8%; B, 28.1%; C, 3.1%).

There is convincing evidence supporting the use of gastroprotective strategies in at-risk patients treated with NSAIDs, which has been adopted by different guidelines worldwide [90,91]. Patients at increased GI risk can be identified by a history of complicated or uncomplicated ulcers, advancing age (patients older than 60 to 65 years, depending on guidelines), use of concomitant medication (especially low-dose aspirin or anticoagulants), and the presence of *H. pylori* infection [61]. NSAID-treated OA patients with risk factors can be exposed to inappropriate therapy as a result of not receiving gastroprotective therapy, not being adherent to the prescribed therapy, or getting non-indicated prevention strategies. Diverse studies with different methodological approaches in different cohorts of patients have reported important findings in this regard. Very low rates of prescription of gastroprotective therapies according to national or international guidelines have been reported, although these rates have increased progressively [92-96]. In the Netherlands, correct prescription rose from 6.9% in 1996 to 39.4% in 2006 in high-risk NSAID users, whereas over-prescription rose from 2.9% to 12.3% [97].

Similar rates were reported in a cross-sectional study of patients prescribed NSAIDs in the United States of America, where only 27.2% of high-risk patients were prescribed a gastroprotective compound according to guidelines. Among patients from VA hospitals with at least two risk factors, adherence to guidelines was 39.7%; among those with three risk factors, adherence was 41.8%. The likelihood of adherence was further decreased if they were prescribed NSAIDs for ≥90 days [98]. Review of medical charts in one large cross-sectional study (n = 17,105) of OA patients found that, in over half of the population examined, NSAID prescriptions did not follow guidelines. Specific areas, where the recommendations were not followed or were overlooked, were in patients with both high GI and CV history (74% inappropriate) and in those with a high GI risk alone (49% inappropriate). However, other recommendations were followed. The study showed high rates of PPI co-prescription with ns-NSAIDs in patients with increased GI risk. However, half of patients with low GI risk and no CV history were still treated with ns-NSAIDs plus a PPI or a COX-2 selective NSAID, contrary to current guidelines [54]. A recent study in Canada has reported that concordance with guideline recommendations increased for celecoxib and decreased for ns-NSAIDs after rofecoxib withdrawal, whereas co-prescription of gastroprotective agents with ns-NSAIDs remained suboptimal, with only 45.6% of at-risk patients receiving these drugs [99].

Adherence by patients to the prescribed drug is another problem. Early reports showed that over one third of patients did not take the gastroprotective agents as prescribed [94]. More recent studies reported similar or better rates for prescription lasting <3 months [100,101], but others reported much lower adherence rates [92,93]. Appropriate prescription and optimal adherence are important for NSAID users; evidence indicates that patients with risk factors who do not receive or follow appropriate prevention strategies have an increased risk of GI complications [100,102]. A recent study involving three European databases found that, among NSAID treated patients with low adherence (<20% of the time with gastroprotection), the odds ratio (OR) was 2.39 (95% CI, 1.66–3.44) for all upper GI events and 1.89 (95% CI, 1.09–3.28) for upper GI bleeding alone when compared to patients who had high levels of adherence (>80% of the time on NSAIDs with gastroprotection) [96]. This increased risk among patients with low adherence was also found in high-risk patients who received a COX-2 selective inhibitor alone whereas guidelines recommend combination of a COX-2 selective inhibitor with a PPI [103].

**Statement 3a:***The efficacy of ns-NSAIDs and COX-2 selective inhibitors in pain is comparable in patients with OA.***Level of Agreement:** Strong Agreement (vote statement: a, 87.5%; b, 12.5%). **Level of Evidence:** A (vote grade: A, 87.5%; B, 9.4%; C, 3.1%).

**Statement 3b:***The efficacy of ns-NSAIDs and COX-2 selective inhibitors in pain is comparable in patients with rheumatoid arthritis* (RA)*.***Level of Agreement:** Strong Agreement (vote statement: a, 84.4%; b, 9.4%; c, 6.3%). **Level of Evidence:** A (vote grade: A, 87.5%; B, 9.4%; C, 3.1%).

The discussion about whether ns-NSAIDs or COX-2 selective inhibitors should be preferred in patients with RA or OA is usually dominated by the possible GI and CV events. Although these adverse effects may have dramatic manifestations, they occur only in a minority of patients. Other issues, like tolerability, adherence to therapy, and cost/price of the drug, may also play a role. However, and perhaps of primary importance, the analgesic and anti-inflammatory effect is crucial for patients suffering from pain due to RA or OA.

In at least five high quality RCTs, a comparable effectiveness has been shown:

1. Different doses of celecoxib (100, 200, and 400 mg/day) were all comparable to naproxen (1,000 mg/day), and superior to placebo, in a 12-week study in patients with RA [104];
2. Celecoxib (200 mg/day) was as effective as diclofenac (150 mg/day) in the long-term management of RA [105];
3. Etoricoxib (60 to 90 mg/day) was as effective as diclofenac (150 mg/day), in RA and OA patients [106];
4. Celecoxib (200 mg/day) was as effective as naproxen 1,000 mg/day in patients with knee OA [107];
5. In the Celecoxib versus Omeprazole and Diclofenac in Patients with Osteoarthritis and Rheumatoid arthritis (CONDOR) study, no difference in effectiveness was found between celecoxib (400 mg/day) and diclofenac (150 mg/day) in RA and OA patients [108].
6. In other studies, rofecoxib was as effective as naproxen [109], and lumiracoxib as diclofenac [110]. However, neither rofecoxib nor lumiracoxib are now available. In fact, all these studies clearly show that ns-NSAIDs and COX-2 selective inhibitors have comparable efficacy, apparent in functioning and disability, as well as in pain.

It is worth emphasizing that, although these drugs seem to have – as a class and at a population-based level – a comparable effect, individual treatment responses may be variable and dose-dependent. Thus, in daily practice the choice between ns-NSAIDs and COX-2 selective inhibitors will depend on the CV and GI risk profile, drug-tolerability, clinical experience of the physician with the given drug, cost/price, and pharmaco-economic considerations, as well as individual patient response.

### GI risks of NSAIDs

**Statement 4:***NSAID use is associated with increased risk of adverse events throughout the entire GI tract; this is associated with substantial mortality.***Level of Agreement:** Strong Agreement (vote statement: a, 75.0%; b, 21.9%; c, 3.1%). **Level of Evidence:** A (vote grade: A, 87.5%; B, 12.5%).

The GI adverse effects of NSAIDs have been well documented in several studies [111-115], meta-analyses [116-118], and Cochrane reviews [119]. The majority of these studies have reported adverse events in the upper GI tract. The meta-analysis by Ofman et al. [118], which reviewed severe upper GI complications in almost 800,000 patients taking oral NSAIDs for at least 4 days, showed an OR of 5.36 (95% CI, 1.79–16.1) from 16 RCTs (versus placebo), RR of 2.7 (95% CI, 2.1–3.5) from 9 cohort studies, and OR of 3.0 (95% CI, 2.5–3.7) from 23 case control studies for severe upper GI complications, which included perforations, clinically relevant ulcers, and bleeding [118].

Similarly, low-dose (≤325 mg daily) aspirin is associated with major upper GI bleeding: the RR was 2.1 (95% CI, 1.6–2.7) in the meta-analysis by McQuaid and Laine [120] and 2.6 (95% CI, 2.2–2.9) in the large Danish cohort study, carried out by Sørensen et al*.* [121]. Lanas et al. [122] recently reported an OR of 1.55 (95% CI, 1.27–1.90) and found that PPI use reduced the risk of major GI bleeding (OR, 0.34; 95% CI, 0.21–0.57).

A predictable and consistent GI blood loss has been shown in healthy volunteers taking ibuprofen (800 mg t.i.d.) [115]. Bleeding was observed in 27/31 subjects (87%) and averaged 4.5 to 5.0 mL/day (SD, 12; range, 0–65 mL/day) with an onset of 3 to 5 days after starting the drug [115].

In the recent CONDOR study, a large, prospective RCT (n = 4,400), the cumulative proportion of patients with adjudicated, clinically significant events throughout the GI tract was significantly greater in those patients taking diclofenac in combination with omeprazole, despite the partial protection provided by the PPI in the upper GI tract, than in patients taking celecoxib [108].

The effect of NSAID use on mortality has been less well studied and a figure of 16,500 deaths in the US has been widely quoted since the original estimate was published [123]. However, in a more recent report of results from two concurrent Spanish studies [114], the incidence of hospital admission due to major GI events of the entire (upper and lower) GI tract was 121.9 events/100,000 person/years and admissions for upper GI complications were six-fold higher than for lower GI tract events. Mortality rates attributed to NSAIDs/low-dose aspirin use were substantial, at 21.0 and 24.8 cases/million people, respectively, and up to one-third of all NSAID/aspirin deaths could be attributed to low-dose aspirin use. Mortality rates associated with either major upper or lower GI events were similar, 5.57% (95% CI, 4.9–6.7) and 5.62% (95% CI, 4.8–6.8), respectively. However, upper GI events were more frequent. Moreover, three further studies reported mortality figures, which range from 3.8 to 11% in patients with peptic ulcer bleeding associated with NSAID use [124-126]. Furthermore, while the adverse events associated with NSAID use can lead to mortality, this could be due to associated causes other than GI adverse events (e.g., CV events) [127].

**Statement 5:***NSAID-induced adverse events in the lower GI tract are not prevented by PPIs.***Level of Agreement:** Strong Agreement (vote statement: a, 75.0%; b, 18.8%; c, 3.1%; d, 3.1%). **Level of Evidence:** B (vote grade: A, 43.8%; B, 56.3%).

While it is clear that PPIs reduce the development of peptic ulcer and ulcer complications in patients taking NSAIDs and/or aspirin, their beneficial effect, which is related to their antisecretory activity [128], is not expected beyond the duodenum [30]. The appreciation that NSAID-associated GI damage does extend beyond the duodenum dates back to the early 90’s, when a few observational studies and the first large RCT prevention study (i.e., the Misoprostol Ulcer Complication Outcomes Safety Assessment (MUCOSA) trial) were published [129]. In the more recent Vioxx™ Gastrointestinal Outcomes Research (VIGOR) trial, more than 40% of the NSAID-related events occurred in the lower (i.e., small bowel and colon) GI tract [130]. A systematic review [131] of 47 studies (18 randomized, 14 case-control, 8 cohort, and 7 before/after studies) found that patients taking ns-NSAIDs had significantly more adverse effects versus no NSAID use in 20/22 lower GI integrity studies (dealing with permeability, inflammation, and microscopic lesions), 5/7 visualization studies, 7/11 bleeding studies (OR, 1.9–18.4 in case-control studies), 2/2 perforation studies (OR, 2.5–8.1), and 5/7 diverticular disease studies (OR, 1.5–11.2). As reported in the Spanish studies (described under Statement 4), over the past decade there has been a progressive change in the overall picture of GI events leading to hospitalization, with a clear decreasing trend in upper GI events and a slight but significant increase in lower GI events [41].

The availability of video capsule endoscopy has allowed a precise quantification of the incidence and characterization of small intestinal damage. Indeed, available studies [132,133] show that about 75% of NSAID users display intestinal mucosal injury, with most denuded areas identified in the proximal part of the small bowel and all ulcers identified in the distal part [134].

In healthy volunteers [133,135,136] and patients [132], omeprazole did not prevent NSAID-associated intestinal damage, evaluated by video capsule and/or fecal calprotectin measurement. The failure of PPIs to protect the small bowel is due to the fact that NSAID-enteropathy is not a pH-dependent phenomenon [30]. Indeed, although inhibition of mucosal prostaglandin synthesis with NSAID use occurs along the entire digestive tract, there are significant differences between the distal and proximal GI tract in the concurrence of other pathogenic factors that may add to mucosal damage. Among the most evident are the absence of acid (which plays a pivotal role in upper GI damage) and the presence of bacteria and bile in the intestine, which may trigger specific NSAID-related pathogenic mechanisms in the distal GI tract [137]. Some recent experimental evidence [138] suggests that PPIs might actually worsen the NSAID intestinal damage by inducing dysbiosis, an adverse event repeatedly described in humans [139,140].

Like non-selective compounds, COX-2 selective NSAIDs damage the small bowel, but the frequency and severity of events are generally lower. Indeed, a systematic review found that COX-2 selective inhibitors had significantly less effect versus ns-NSAIDs in 3/4 GI integrity studies, one endoscopic study (RR mucosal breaks, 0.3), and two randomized studies (RR lower GI clinical events, 0.5; hematochezia, 0.4) [131]. The better intestinal tolerability of the selective agent, celecoxib, persisted even when the ns-NSAID was combined with a PPI [135,136]. In addition, switching RA patients on long-term ns-NSAIDs to celecoxib resulted in a significant reduction of small bowel injury [141]. While two large outcome studies (the VIGOR and CONDOR trials) showed a reduced risk of more serious events in the lower or the whole GI tract, for rofecoxib and celecoxib, respectively [108,130], this benefit was not confirmed for etoricoxib in the Multinational Etoricoxib versus Diclofenac Arthritis Long-term (MEDAL) program [142].

The impact of upper GI tract adverse effects can be mitigated by the use of PPIs. The lack of efficacy of these agents in preventing lower GI tract damage is however an unmet clinical need that requires addressing. The use of non acidic COX-2 selective inhibitors, such as celecoxib, may help to reduce the risk of damage throughout the entire GI tract, but much work is needed to define the best preventive strategy for the lower GI tract in NSAID users.

**Statement 6:***Celecoxib is associated with fewer adverse events throughout the entire GI tract compared to ns-NSAIDs.***Level of Agreement:** Strong Agreement (vote statement: a, 62.5%; b, 18.8%; c, 18.8%). **Level of Evidence:** A (vote grade: A, 46.9%; B, 43.8%; C, 9.4%).

A meta-analysis of RCTs showed that COX-2 selective inhibitors were associated with significantly fewer gastro-duodenal ulcers (RR, 0.26; 95% CI, 0.23–0.30) and clinically important ulcer complications (RR, 0.39; 95% CI, 0.31–0.50) than ns-NSAIDs [143]. Other studies have also shown that celecoxib is as safe to the upper GI tract as ns-NSAIDs plus a PPI [144].

It is now well known that NSAIDs induce mucosal damage to the small and large bowel, including inflammation, erosions, ulcers, bleeding, perforation, and obstruction [61,145]. Epidemiological studies have reported an increased risk of lower GI bleeding and perforation with NSAID and aspirin use [146,147]. More recently, biochemical and endoscopy capsule studies have shown a high frequency of mucosal inflammation and mucosal breaks in both healthy subjects [133] and patients taking NSAIDs [132]. Although PPIs are increasingly used to prevent NSAID-related GI adverse events, they do not protect from lesions beyond the duodenum (see Statement 5). This may explain the increasing number of hospitalizations due to complications of the lower GI tract, seen in some studies, whereas the corresponding numbers for upper GI complications are decreasing (see Statement 4) [41].

Capsule endoscopy studies [135,136] have demonstrated that, compared to ns-NSAIDs plus a PPI, celecoxib alone was associated with less mucosal damage of the small bowel in healthy volunteers. In the 6-month, double-blind, randomized CONDOR trial, in patients with OA or RA at increased GI risk [108], fewer (0.9%) subjects receiving celecoxib (200 mg b.i.d) met the criteria for the primary endpoint (clinically significant upper and lower GI events) compared to those receiving diclofenac (75 mg b.i.d.) plus omeprazole (3.8%; hazard ratio [HR] = 4.3; 95% CI, 2.6–7.0). Also, fewer patients taking celecoxib withdrew early because of GI adverse events compared to patients taking diclofenac plus omeprazole. Most of the outcome events were a drop in hemoglobin ≥2 g/dL attributed to identified lesions from the upper GI tract or to presumed small bowel lesions. These data have been confirmed in a recent 6-month randomized, open-label, blinded endpoint study that measured clinical outcomes throughout the GI tract [148]. Celecoxib use was associated with a lower risk of clinically significant upper and lower GI events than ns-NSAIDs (OR, 1.82; 95% CI, 1.31–2.55) [148]. However, a recent epidemiological study carried out in Taiwan has shown celecoxib to be associated with an increased risk of complications in the lower GI tract, similar to other NSAIDs [149]. These data need to be confirmed, since epidemiological studies are inevitably associated with different biases and confounding factors that may be difficult to control.

A relevant question is whether data on the safety of celecoxib in the lower GI tract can be extrapolated to other COX-2 selective inhibitors, since NSAID-associated mucosal damage in the lower GI tract depends not only on COX-1/COX-2 inhibition, but also on the physicochemical properties and the entero-hepatic circulation of individual NSAIDs [30].

In a 28-day trial conducted in healthy volunteers, Hunt et al. [150] found that fecal blood loss with etoricoxib (120 mg o.d.) was similar to placebo and significantly lower than that found in patients treated with ibuprofen (800 mg t.d.s.). However, the MEDAL program [142] did not confirm a decrease in lower GI complications with etoricoxib versus diclofenac.

**Statement 7:***The combination of celecoxib plus low-dose aspirin is associated with a lower risk of adverse events in the upper GI tract, as compared with ns-NSAIDs plus low-dose aspirin.***Level of Agreement:** Strong Agreement (vote statement: a, 43.8%; b, 28.1%; c, 6.3%; d, 9.4%; e, 3.1%; f, 9.4%). **Level of Evidence:** B (vote grade: A, 25.0%; B, 40.6%; C, 25.0%; D, 3.1%; E, 6.3%).

It is well established that low-dose aspirin exerts a life-saving antithrombotic effect, particularly in secondary prevention [151]. However, a large cohort study [121] and a meta-analysis of 35 RCTs, including 87,581 patients [122], have clearly shown that its use is associated with an increased risk of GI bleeding. This risk is further increased when aspirin is combined with clopidogrel or anticoagulants [122] and appears to be independent of the formulation of aspirin used (e.g., buffered or enteric coated) [152].

The well-known risk of upper GI events associated with the use of ns-NSAIDs is significantly enhanced by concomitant use of low-dose aspirin, a quite frequent clinical situation in older patients having both OA and CV co-morbidities [153,154]. A large, hospital-based, case-control study, performed in Spain, found that adding low-dose aspirin to ns-NSAIDs, increased the risk of upper GI bleeding by more than two-fold [155]. Similarly, a Canadian retrospective cohort study found a 62% increase in hazard ratio of hospitalization for GI events in patients taking these combinations, although the combination of celecoxib with low-dose aspirin was associated with a lower risk (HR, 0.62; 95% CI, 0.48–0.80) than the association of ns-NSAIDs plus low-dose aspirin [156].

When COX-2 selective inhibitors were examined as a class, conflicting results were reported by different studies. In the case of three epidemiological studies, one (a hospital-based, case-control) found a similar RR of upper GI bleeding when low-dose aspirin was combined with COX-2 selective inhibitors or ns-NSAIDs [155], while the others (nested case-control studies, performed in databases from the UK) observed an increased RR of upper GI complications when ns-NSAIDs or COX-2 selective agents were used together with an antiplatelet agent [157,158]. However, in a meta-analysis of four RCTs including 17,276 patients, the RR for perforation, ulcer, and bleeding between the combination low-dose aspirin/COX-2 selective inhibitors and low-dose aspirin/ns-NSAIDs was 0.72 (95% CI, 0.62–0.95) [143]. However, these were *post hoc* analyses and not randomised comparisons, which suggest a possible bias by patient selection.

As expected, the analysis of the risk associated with the combination of low-dose aspirin with individual COX-2 selective inhibitors also yielded different results. Indeed, with the exception of the Celecoxib Long-Term Arthritis Safety Study (CLASS) trial (where high doses, i.e., 800 mg/day, of the drug were employed) [159], all the available studies (be they epidemiological or RCTs) [156,160-162], as well as a meta-analysis of 31 RCTs (n = 39,605) [163], point to a lower upper GI risk of low-dose aspirin in combination with celecoxib than with the same antiplatelet agent combined with an ns-NSAID. However, it must be clear that low-dose aspirin potentiates the GI risk of either a selective COX-2 inhibitor or ns-NSAID and that, in patients with high GI risk, these combinations may still be harmful and gastroprotection with a PPI seems appropriate and beneficial [31].

In contrast, in the MEDAL program, the pre-specified pooled intent-to-treat analysis of the three RCTs (i.e., Etoricoxib versus Diclofenac sodium Gastrointestinal tolerability and Effectiveness trial (EDGE)-I, EDGE-II, and MEDAL), comparing etoricoxib with diclofenac in an overall population of 11,418 OA/RA patients taking low-dose aspirin the HR for overall upper GI clinical events was 0.78 (95% CI, 0.60–1.1), indicating a lack of significant difference between the two anti-inflammatory drugs [164]. Consistent with this finding, the cumulative rate of patient discontinuation due to clinical and laboratory upper GI adverse events was also not statistically different in the two treatment arms [165]. No formal studies have evaluated the GI risk of dual antiplatelet therapy in patients taking either ns-NSAIDs or coxibs.

Recent video capsule studies have shown that low-dose aspirin is also harmful to the small intestine [166-168]. The combination of aspirin with ns-NSAIDs would likely be more damaging. However, studies providing such evidence are lacking and, more importantly, the clinical relevance of these mucosal lesions need to be defined in the context of serious and life-threatening outcomes such as bleeding and perforation.

### CV risk of NSAIDs

**Statement 8:***The risk of CV events associated with celecoxib use is similar to that associated with the use of most ns-NSAIDs.***Level of Evidence:** Strong Agreement (vote statement: a, 68.8%; b, 15.6%; c, 9.4%; d, 6.3). **Level of Evidence:** A (vote grade: A, 53.1%; B, 40.6%; C, 6.3%).

Although OA is not apparently an independent risk factor for CV disease, many OA patients are elderly and concomitant CV disease is not uncommon. In a nested case-control study (n ≥1,400,000) by Graham et al. [169], an increased CV risk was associated with rofecoxib as well as ns-NSAIDs. In RCTs, rofecoxib demonstrated a higher incidence of CV adverse events than naproxen in non-aspirin users with RA [109] or than placebo in patients with colorectal adenomas (Adenomatous Polyp Prevention on Vioxx™ (APPROVe) trial) [170]. For celecoxib, an increase in CV events was noted in patients with colorectal cancer when compared with placebo [171]. However, in these trials, the absolute numbers were low. In a RCT in Alzheimer’s disease, CV events were higher with naproxen compared to celecoxib or placebo [172]. In the CLASS study performed in OA and RA patients, there was no difference in CV events between celecoxib (400 mg b.i.d.) and ns-NSAIDs [173]. In the MEDAL trials, etoricoxib showed a similar cumulative incidence of thrombotic CV events to diclofenac [106]. A large meta-analysis of cohort and nested case-control studies also found an increased risk of CV events for all ns-NSAIDs and COX-2 selective inhibitors [46]. Other meta-analyses concluded that COX-2 selective agents and ns-NSAIDs have similar CV risk [47,174,175]. A recent network meta-analysis, which aimed to compare the CV risk of ns-NSAIDs and COX-2 selective inhibitors, found a similar CV risk between these two classes of anti-inflammatory compounds [48]. Naproxen appeared to be the least harmful, but this advantage has to be weighed against GI toxicity. It also must be considered that the absence of an increased CV risk observed in RCTs and meta-analysis with naproxen, when compared to placebo, was based on a high naproxen dose (500 mg b.i.d) [47].

Putting the CV risk in OA patients into context, the incidence of the GI risk is higher than the CV risk, and COX-2 selective inhibitors have a lower GI risk than ns-NSAIDs. Taking all the available data from clinical trials, meta-analyses, and cohort studies into account, the overall CV risk is increased for both ns-NSAIDs and COX-2 selective inhibitors. For each COX-2 selective inhibitor, however, the reduction of complicated upper GI events was numerically greater than any increase in Antiplatelet Trialists’ Collaboration events (fatal or non-fatal myocardial infarction or stroke, or vascular death) [176]. There is no evidence of major differences between ns-NSAIDs and COX-2 selective inhibitors. Hitherto, there is no published RCT which has been specifically designed to compare CV risk between ns-NSAIDs and COX-2 selective inhibitors. Results of the Prospective Randomized Evaluation of Celecoxib Integrated Safety versus Ibuprofen or Naproxen (PRECISION) study will provide important data on the comparative CV safety of celecoxib, ibuprofen, and naproxen. It is worthwhile mentioning that this trial, performed in a high CV risk population, is the first study specifically designed to assess the CV safety of anti-inflammatory drugs [177]. Unfortunately, the trial has been delayed because of slow enrollment and full results are not expected until 2016 [178].

**Statement 9:***COX-2 selective inhibitors do not interfere with the antiplatelet effect of low-dose aspirin*. **Level of Agreement:** Strong Agreement (vote statement: a, 65.6%; b, 21.9%; c, 6.3%; d, 6.3%). **Level of Evidence:** A (vote grade: A, 77.4%; B, 16.1; C, 3.2%; D, 3.2%).

The benefits of low-dose aspirin use in secondary prevention clearly outweigh the risk [151]. However, this is not the case for primary prevention [179], where recommendations for aspirin use should be individualized, taking into account the balance between benefits and risks, as well as individual patient values and preferences [180]. Given that aspirin is a life-saving drug, discontinuing it or not adhering to the correct administration schedule enhances the risk of CV and cerebral events by more than three-fold [181,182]. This risk was magnified by up to 90-fold in patients with intracoronary stents [182].

Ns-NSAIDs, being COX-1-inhibitors, all impair thromboxane A_2_ synthesis and, as a consequence, platelet aggregation, although the magnitude and duration of this effect varies amongst the different compounds [183]. With the exception of diclofenac [184,185] and meloxicam [186], almost all ns-NSAIDs can interfere with the anti-aggregant effect of aspirin [185,187-189].

While the pharmacodynamic, negative interaction between ns-NSAIDs and low-dose aspirin has been clearly established by studies in healthy volunteers and patients, the clinical consequences of such interaction are still not definitely ascertained. Indeed, available epidemiological studies provided conflicting results, with only three out of six reports showing a reduction of the cardio-protective effect of aspirin [190-195]. However, the few RCTs available are consistent in their findings that ns-NSAID use does worsen the CV outcome in patients taking low-dose aspirin. Indeed, in the Physicians’ Health Study, the regular use of these agents was associated with an increased risk (RR, 2.86; 95% CI, 1.25–6.56) of acute recurrent myocardial infarction [196]. Similarly, in the Therapeutic Arthritis Research and Gastrointestinal Event Trial (TARGET), designed to assess the GI and CV safety of lumiracoxib versus naproxen and ibuprofen, a *post hoc* subgroup analysis of high CV risk patients (n = 3,042) taking low-dose aspirin (60%) found a higher CV event rate in ibuprofen users compared to lumiracoxib users (1.48 versus 0.85 events per 100 patient/years) [197]. Besides the CV harm, concomitant administration of ns-NSAIDs and low-dose aspirin can be followed by stroke recurrence in patients with prior cerebrovascular events [188].

COX-2 selective NSAIDs spare platelet COX-1 activity [198] and do not affect platelet aggregation [184,198-201] or bleeding time [200]. Similarly, COX-2 selective inhibitors do not interfere with the anti-aggregant activity of low-dose aspirin both in healthy subjects [184,186,187,198] and patients with coronary heart disease [202,203]. Consistent with these results, *in vitro* studies on human platelets have shown that a low affinity for COX-1 and a high degree of COX-2 selectivity confers a low potential to block aspirin inhibition of platelet COX-1 [204].

Taking all the above lines of evidence into account, COX-2 selective inhibitors should represent the anti-inflammatory drugs of choice for patients taking low-dose aspirin for CV or cerebrovascular prevention [205], provided the anti-inflammatory therapy is deemed necessary and cannot be avoided with alternative therapies. It should be emphasized, however, that the European Medicines Agency’s (EMA) Committee for Medicinal Products for Human Use stated - in 2005 - that “*COX-2 inhibitors must not be used in patients with established ischemic heart disease and/or cerebrovascular disease (stroke)*” [206]. Therefore, the benefits (i.e., lack of blunting of aspirin protection and the anti-inflammatory/analgesic activity) should be balanced against the possible CV risks, which ultimately depend on individual patients’ clinical characteristics and co-medications.

## Discussion

Current guidelines on NSAID use have been developed by rheumatologists [60,65], gastroenterologists [57-59,61], cardiologists [64,70], or multidisciplinary teams of experts [62,63,66,68,69]. Rheumatologists were first concerned with safety, thus recommending paracetamol (acetaminophen) as a first-line analgesic. Gastroenterologists dealt mainly with GI risk factors and gastroprotection, emphasizing how misused and underused it is, while cardiologists were worried about CV safety and suggested naproxen use in patients with CV risk factors. Some multidisciplinary consensus papers discussed both GI and CV risks and put forward evidence-based proposals on how to balance the benefits and risks of anti-inflammatory therapy [66,68,69]. Despite this, some important issues have been left unsettled, partly because sufficient evidence was not available at the time of guideline drafting.

Navigating through the different GI and CV risk factors and balancing them with the potential benefits of NSAID therapy is a difficult task. This is why a team of 34 experts, from five different disciplines, gathered together to critically examine and grade the current evidence with the aim of achieving a consensus on how to best manage complex patients with high GI and/or CV risks. The Consensus statements and their comparison with those of previous guidelines [8,26,27,64,66-68,32] are summarized in Table 2.

**Table 2:** **Comparison between the statements of this expert consensus with related statements issued by different guidelines**

**Statement of this expert consensus**		**EULAR guidelines** ** (2005) [** 26 **]**	**Joint ACCF/ACG/AHA and AHA guidelines** ** (2007–2008) [** 64 **,** 66 **]**	**OARSI guidelines** **(2008) [** 27 **]**	**ACR guidelines** ** (2008) [** 8 **]**	**Intl working party** ** (2008) [** 67 **]**	**Canadian consensus** ** (2009) [** 68 **]**	**ACG guidelines** ** (2009) [** 32 **]**
1	*OA impacts quality and quantity of life; it should therefore be treated appropriately*	No statement	No statements	The optimal management of OA requires a combination of non-pharmacological and pharmacological treatment modalities	No statement	No statement	No statement	No statement
2	*Many OA patients requiring NSAID therapy are not being treated appropriately according to their GI risk profile*	No statement	No statements	No statement	No statement	No statement	No statement	No statement
3a	*The efficacy of ns-NSAIDs and COX-2 selective inhibitors in pain is comparable in patients with OA*	NSAIDs, at the lowest effective dose, should be added or substituted in patients who respond inadequately to paracetamol	No statements	No statement	Selective and ns-NSAIDs have comparable efficacy in treating pain and improving function in the treatment of OA and RA pain	No statement	In general, ns-NSAIDs have similar effectiveness in improving pain and function in patients with arthritis	No statement
3b	*The efficacy of ns-NSAIDs and COX-2 selective inhibitors in pain is comparable in patients with RA*	See above	No statements	No statement	*See above*	No statement	COX-2 inhibitors are as effective as ns-NSAIDs in improving pain and function in patients with arthritis	No statement
4	*NSAID use is associated with increased risk of adverse events throughout the entire GI tract; this is associated with substantial mortality*	In patients with increased GI risk, ns-NSAIDs plus a gastroprotective agent, or a selective COX-2 inhibitor (coxib) should be used	No specific statements, but the guidelines assume that NSAIDs increase the risk of upper GI complications	In patients with symptomatic hip or knee OA, NSAIDs should be used at the lowest effective dose, but their long-term use should be avoided if possible	NSAIDs are associated with GI adverse events, including peptic ulcer disease, gastritis, esophagitis, and their complications	No specific statement, but the document assumes that NSAIDs increase the risk of upper GI complications	Aspirin and ns-NSAIDs increase the risk of upper GI complications. Aspirin and ns-NSAIDs increase the risk of small and large bowel bleeding and other complications	Patients requiring NSAID therapy who are at high risk (e.g., prior ulcer bleeding or multiple GI risk factors) should receive alternative therapy, or if anti-inflammatory treatment is absolutely necessary, a COX-2 inhibitor, and co-therapy with misoprostol or high-dose PPI
5	*NSAID-induced adverse events in the lower GI tract are not prevented by PPIs*	In patients with increased GI risk, ns-NSAIDs plus a gastroprotective agent, or a selective COX-2 inhibitor (coxib) should be used	PPIs are the preferred agents for the therapy and prophylaxis of NSAID- and aspirin-associated GI injury	In patients with increased GI risk, either a COX-2 selective agent or a ns-NSAID with coprescription of a PPI or misoprostol for gastroprotection may be considered	If a patient and provider agree to utilize an NSAID for arthritis pain relief, and the patient has risk factors for GI bleeding, then the patient should be treated concomitantly with either misoprostol or a PPI	No specific statement and no mention of the lower GI tract	PPI therapy reduces the risk of ns-NSAID associated endoscopic ulcer disease, but there is less evidence for a reduction in bleeding events. In patients with prior GI bleeding, the combination of a PPI and a COX-2 inhibitor reduces the risk of upper GI bleeding from that of COX-2 inhibitors alone	No statement
6	*Celecoxib is associated with fewer adverse events throughout the entire GI tract compared to ns-NSAIDs*	In patients with increased GI risk, ns-NSAIDs plus a gastroprotective agent, or a selective COX-2 inhibitor (coxib) should be used	No specific statement, but the guidelines assume that coxibs are safer than ns-NSAIDs for the upper GI tract	In patients with increased GI risk, either a COX-2 selective agent or a ns-NSAID with coprescription of a PPI or misoprostol for gastroprotection may be considered	No specific statement	Coxibs considered safer than ns-NSAIDs to the upper GI tract	Compared to ns-NSAIDs, COX-2 inhibitors are associated with a lower risk of upper GI bleeding	COX-2 inhibitors are associated with a significantly lower incidence of gastric and duodenal ulcers when compared to traditional NSAIDs
7	The combination of celecoxib plus low-dose aspirin is associated with a lower risk of adverse events in the upper GI tract, as compared with ns-NSAIDs plus low-dose aspirin.*	No statement	As the use of any NSAID, including COX-2 selective agents and over-the-counter doses of traditional NSAIDs, in conjunction with low-dose aspirin, substantially increases the risk of ulcer complications, a gastroprotective therapy should be prescribed for at-risk patients	No statement	If a patient is taking aspirin for cardioprotective benefit, then selective and ns-NSAIDs should be avoided	All patients should be given a PPI if on aspirin and taking NSAIDs.	The risk of GI bleeding is increased when aspirin is co-prescribed with ns-NSAIDs compared to NSAIDs alone. The risk of GI bleeding is increased when aspirin is co-prescribed with COX-2 inhibitors compared with COX-2 inhibitors alone	The GI benefit of COX-2 inhibitors is negated when the patient is taking concomitant low-dose aspirin.
						Naproxen recommended as NSAID of choice		
								Patients for whom anti-inflammatory analgesics are recommended, who also require low-dose aspirin therapy for CV disease, can be treated with naproxen plus misoprostol or a PPI
8	*The risk of CV events associated with celecoxib use is similar to that associated with the use of most ns-NSAIDs*	No statement	The AHA guidelines assume that coxibs carry the highest CV risk and recommend the use of naproxen as the drug of choice for patients with CV risk	NSAIDs, including both non-selective and COX-2 selective agents, should be used with caution in patients with CV risk factors	Selective NSAIDs might pose increased CV risk compared with ns-NSAIDs through several potential mechanisms. A systematic review of observational studies suggests that celecoxib, in commonly used doses, does not significantly increase CV risk. It is likely that higher doses, particularly when administered twice daily, increase the CV risk	Use of coxibs considered inappropriate; use of naproxen considered appropriate	COX-2 inhibitors increase the risk of coronary heart disease events; non-naproxen ns-NSAIDs increase the risk of coronary heart disease events; naproxen is associated with a lower risk of coronary heart disease events than other ns-NSAIDs and COX-2 inhibitors	The lowest possible dose of celecoxib should be used in order to minimize the risk of CV events. Patients at moderate GI risk who also are at high CV risk should be treated with naproxen plus misoprostol or a PPI. Patients at high GI and high CV risk should avoid using NSAIDs or coxibs. Alternative therapy should be prescribed
9	*COX-2 selective inhibitors do not interfere with the antiplatelet effect of low-dose aspirin*	No statement	Evidence indicates that ibuprofen, but not rofecoxib (a COX-2 inhibitor), interferes with aspirin’s ability to irreversibly inactivate COX-1	No statement	Selective NSAIDs do not appear to have relevant drug–drug interactions with the anticoagulant effect of aspirin	No specific statement or comment	No statement	No statement

*This does not preclude the use of PPIs for gastroprotection. ACCF, American College of Cardiology Foundation; ACG, American College of Gastroenterology; ACR, American College of Rheumatology; AHA, American Heart Association; CV, Cardiovascular; EULAR, European League Against Rheumatism; GI, Gastrointestinal; NSAIDs, Non-steroidal anti-inflammatory drugs; ns-NSAIDs, Non-selective NSAIDs; OA, Osteoarthritis; OARSI, Osteoarthritis Research Society International; PPI, Proton pump inhibitors; RA, Rheumatoid Arthritis; RCT, Randomized clinical trial.

On the basis of this consensus, before starting anti-inflammatory therapy, the real need for it should be carefully assessed and the CV risks (that are not easily modifiable) as well as upper and lower GI risk factors should be quantified. Until now, the evaluation of lower GI risk has been hampered by the lack of knowledge of the corresponding risk factors, which – conversely from those related to upper GI complications – are still poorly understood. Analysis of the data from the MEDAL program (i.e., MEDAL, EDGE-I, and EDGE-II studies) has shown that the risk of a lower GI clinical event with NSAID use seems to be constant over time, and the major risk factors are a prior lower GI event and older age [142]. Colonic diverticula were the most common cause of bleeding in this study, confirming that NSAIDs are independent risk factors for colonic diverticular hemorrhage [207]. Old age (≥65 years) and a recent event were also found to be significant risk factors in epidemiological studies [141,155], which also demonstrated that an increased number of co-morbidities represent an additional risk factor for lower GI complications. In a cross-sectional video capsule study, performed in RA patients under NSAID treatment for more than 3 months, elderly patients and users of acid suppressants (H_2_-receptor antagonists and PPIs) were more likely to develop severe enteropathy [208]. A recent *post hoc* analysis of the CONDOR trial [209] showed that baseline C-reactive protein levels, history of gastritis and of GI intolerance, *H. pylori* infection, old age, and body mass index were all associated with clinically significant blood loss in OA patients treated with NSAIDs.

The better upper GI safety of COX-2 selective agents over ns-NSAIDs is well established [150,163,210-212]. However, their individual lower GI tolerability is less well evidenced and appears to differ. Indeed, while the serious lower GI events with rofecoxib (withdrawn from the market) were significantly reduced in comparison with ns-NSAIDs [130], this was not the case for etoricoxib [142]. Likewise, the CONDOR trial [108], by using the novel composite score of clinically significant upper and lower GI events [213], has shown that celecoxib has a better GI safety in the entire GI tract compared to diclofenac plus omeprazole, a finding confirmed by the recent GI-REASONS trial in the real world setting [148]. A recent meta-analysis of 51,000 patients enrolled in 52 RCTs from the celecoxib clinical database showed that, when compared with ns-NSAIDs, celecoxib is associated with a significantly lower risk of all clinically significant GI events throughout the entire GI tract [214]. This superior lower GI tolerability in patients is backed by video capsule studies in healthy volunteers [135,136] and RA patients, where the number and severity of intestinal lesions during ns-NSAID therapy were significantly reduced after switching to celecoxib [141].

Being a COX-2 selective agent, celecoxib displays the same overall CV risk of ns-NSAIDs [45-48]. This was confirmed by a recent meta-analysis of individual participant data from RCTs [215]. This study showed that the vascular risks of high-dose diclofenac, and possibly ibuprofen, are comparable to selective COX-2 agents, whereas high-dose naproxen is associated with less vascular risk than other NSAIDs. This conclusion, however, should be viewed with caution since it was based on non-randomized and indirect comparison between the different compounds. As a consequence, the US Food and Drug Administration Advisory Panel recently felt that the evidence of CV safety is not conclusive enough to warrant a label change, especially pending the results of the PRECISION study [216]. Interestingly, direct comparison of celecoxib (both 200 and 400 mg) with naproxen did not show any difference in CV risk while both rofecoxib and etoricoxib displayed an increased risk [215].

A meta-analysis of RCTs in rheumatologic conditions failed to show an increased CV risk associated with celecoxib compared to placebo [174], while a safety analysis from six RCTs, performed in patients with conditions other than arthritis, provided evidence of a differential CV risk as a function of celecoxib dose, regimen, and baseline CV risk [217]. The risk appeared to be non-significant (HR, 1.1; 95% CI, 0.6–2.0) for the 400 mg q.d. dose, intermediate for the 200 mg b.i.d. dose (HR, 1.8; 95% CI, 1.1–3.1), and high for the 400 mg b.i.d. dose (HR, 3.1; 95% CI, 1.5–6.1) [217]. The recent Coxib and traditional NSAID Trialists meta-analysis [215] also found a trend towards less risk with lower celecoxib doses. Indeed, the vascular effects of celecoxib 200 mg daily (the most widely used regimen) were statistically uncertain. In contrast to ns-NSAIDs, celecoxib does not impair the antiplatelet activity of low-dose aspirin, either alone [187,202] or in combination with clopidogrel [203]. This lack of interference with the antithrombotic action of antiplatelet drugs would make this COX-2 selective agent a suitable anti-inflammatory drug for patients receiving low-dose aspirin for CV or cerebrovascular prevention [205], despite the contrary opinion of the EMA [206]. However, at the time when the Committee for Medicinal Products for Human Use issued its recommendations, much of the current evidence was not yet available. Although no specific data are available, the present recommendations should be valid for patients either on single or dual antiplatelet therapy.

Epidemiological studies have shown that the presence of co-morbidities increases both the GI [218] and CV [219] risk, which are not stable. Previous GI bleeding (recent or remote) put NSAID users at high risk of re-bleeding. NSAID treatment should therefore be avoided but – if mandatory – after *H. pylori* eradication in infected patients, celecoxib plus a PPI are the best evidence-based therapeutic option [31]. Although no current guideline suggests an option for lower GI bleeding, the better upper and lower GI tolerability of celecoxib outlined above does suggest its combination with a PPI is the best strategy for prevention of both upper and lower GI bleeding in high risk patients.

Along the same lines, elevated CV risk is most prominent soon after acute myocardial infarction (AMI), although risk declines with time [220]. Although NSAIDs are contraindicated among patients with established CV disease, many receive NSAID treatment for a short period of time. Even short-term treatment with most NSAIDs has been associated with increased risk of death and recurrent AMI in patients with prior AMI [221]. However, no increased risk was observed for celecoxib users in a population-based cohort of Canadian patients aged 66 years and older, who survived hospitalization for AMI [222]. Therefore, even in patients with high CV risk, celecoxib appears to be the least harmful NSAID, especially since patients will be on low-dose aspirin therapy.

**Figure 1:**
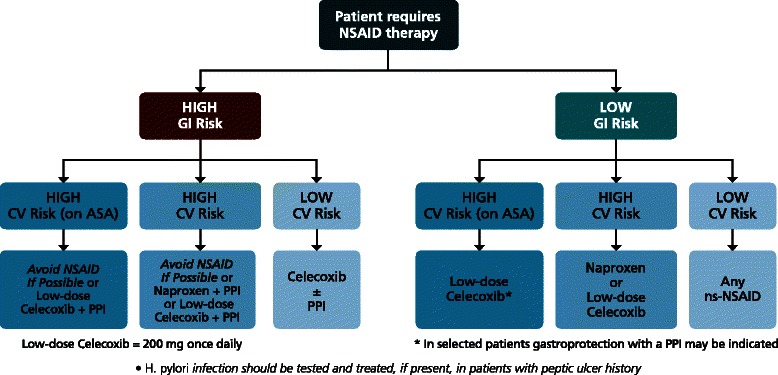
**Algorithm for long-term NSAID therapy according to a patient’s GI and CV risk factors.**

Taking into account the risk/benefit ratio of the available COX-2 selective inhibitors and ns-NSAIDs, celecoxib at lowest approved dose (200 mg once daily, hereinafter referred to as low-dose) appears to be the safest option in patients with concomitant high GI (both upper and/or lower) and CV risks, if NSAIDs are necessary and the patient is on low-dose aspirin. Figure 1 suggests an algorithm for the use of long-term NSAID therapy according to individual GI and CV risk. For patients with both low GI and CV risks, any ns-NSAID alone may be acceptable. For those with low GI and high CV risk, naproxen may be preferred because of the potential lower CV risk compared with other ns-NSAIDs or COX-2 selective inhibitors. Taking into account the current evidence [174,217-224], also low-dose (200 mg), once daily celecoxib may be acceptable. However, in those patients who are on low-dose aspirin, naproxen would impair the anti-aggregant activity of this antiplatelet agent. This interaction is more evident when this NSAID is given before aspirin than when it is given after [189]. COX-2 selective inhibitors, as a class, are therefore indicated as anti-inflammatory agents [70,205], a choice also recommended by the Canadian Cardiovascular Society [70]. Low-dose celecoxib is the preferred agent because of a better CV and GI profile.

In patients with high GI risk, testing for and eradicating *H. pylori* should be considered [33,34,68], but will be insufficient without ongoing gastroprotection. In these patients, if CV risk is low, a COX-2 selective inhibitor alone or ns-NSAID with a PPI appear to offer similar protection from upper GI events. However, according to recent evidence, only celecoxib will reduce mucosal harm throughout the entire (upper and lower) GI tract. This agent should be combined with a PPI in patients at very high risk of upper GI bleeding [31]. When both GI and CV risks are high, the optimal strategy is to avoid NSAID therapy, if at all possible. If the NSAID therapy is deemed necessary, the therapeutic approach will depend on co-medication. If the patient is not on antiplatelet therapy (because, for instance, the benefits of primary prevention have been considered not to outweigh the risks [179]), either naproxen or low-dose celecoxib could be considered, but should be combined with a PPI, while this COX-2 selective agent seems the most appropriate choice in patients on low-dose aspirin. It should be emphasized that the suggested algorithm should be viewed as a “general guidance”, which needs to be tailored to the individual patient, taking into account co-morbidities and co-therapies.

Besides efficacy and safety, costs will also influence therapeutic choices. However, together with medication costs, the economic burden on the healthcare system of NSAID-induced GI or CV events should be taken into account. Prior to the 2008 National Institute for Health and Clinical Excellence (NICE) clinical guideline for the management of OA [225], a number of economic evaluations of COX-2 inhibitors and NSAIDs had been completed, but none included the full range of important adverse events. While most included GI adverse events, none included all of the relevant CV events [226]. The 2008 NICE clinical guideline for OA included an update [227] of their 2001 technology appraisal of COX-2 selective inhibitors and NSAIDs [228]. For the guideline, the economic model was updated to include new available evidence from the CONDOR study. The analysis found that adding a PPI to an ns-NSAID or a COX-2 selective inhibitor was a cost-effective treatment strategy [227,229]. This was the case for patients at relatively low GI risk, as well as for those at high risk. When the CONDOR study became available, the model was adapted to include relative risks of adverse events concerning the lower GI tract [230]. The results of the analysis showed that celecoxib plus a PPI remains a cost-effective strategy for the treatment of OA compared to diclofenac plus a PPI; this is an important new message for clinicians. Indeed, previous guidance recommended that adding a PPI to an ns-NSAID or prescribing a COX-2 inhibitor should only be considered for patients at high risk of GI adverse events, and that use of a PPI in addition to a COX-2 inhibitor would not be cost effective, even for high-risk patients [228].

## Conclusions

NSAIDs are an essential part of our therapeutic armamentarium despite their well characterized GI and CV risk profiles. The time is now ripe for offering the patient with OA the safest and most cost-effective therapeutic option, thus preventing serious adverse events, which could have important QoL and resource use implications. The integration of existing guidelines with the present one, together with a careful evaluation of both GI and CV risk factors, should allow clinicians to correctly manage OA patients without expanding the already growing NSAID epidemic. In the future, the potential chemopreventive effects of NSAIDs/coxibs [231,232] and of low-dose aspirin [233,234] on GI (as well as non-GI) cancers may impact the benefits and risks equation, observed in the arthritic population, and expand the use of these compounds.

## Additional file

Additional file 1:
**NSAID Consensus Group: voting experts and non-voting observers.**

## Abbreviations

AMI: Acute myocardial infarction
CI: Confidence intervals
CONDOR: Celecoxib versus Omeprazole and Diclofenac in Patients with Osteoarthritis and Rheumatoid arthritis
COX: Cyclooxygenase
CV: Cardiovascular
EDGE: Etoricoxib versus Diclofenac sodium Gastrointestinal tolerability and Effectiveness trial
EMA: European Medicines Agency
GI: Gastrointestinal
*H. pylori*: *Helicobacter pylori*
MEDAL: Multinational etoricoxib versus diclofenac arthritis long-term
NICE: National Institute for Health and Clinical Excellence
NSAIDs: Non-steroidal anti-inflammatory drugs
ns-NSAIDs: Non-selective NSAIDs
OA: Osteoarthritis
OR: Odd ratio
PPI: Proton pump inhibitor
PRECISION: Prospective randomized evaluation of Celecoxib integrated safety versus Ibuprofen or Naproxen
QoL: Quality of life
RA: Rheumatoid arthritis
RCT: Randomized clinical trial
RR: Relative risk
TARGET: Therapeutic arthritis research and gastrointestinal event trial
VIGOR: Vioxx™ Gastrointestinal outcomes research
