# Supplementary material for: Safe prescribing of non-steroidal anti-inflammatory drugs in patients with osteoarthritis – an expert consensus addressing benefits as well as gastrointestinal and cardiovascular risks
Source: BMC Med. 2015 Mar 19;13:55. doi: 10.1186/s12916-015-0285-8 (PMC4365808; doi:10.1186/s12916-015-0285-8)
Supplement: Additional file 1: — NSAID Consensus Group: voting experts and non-voting observers. [file 12916_2015_285_MOESM1_ESM.docx]

**Additional file 1** – NSAID Consensus Group: Voting Experts and Non-Voting Observers

| **Non-Voting Chair** | **Gastroenterologists** | **Cardiologists** | **Non-Voting Observers: Pfizer** |
| --- | --- | --- | --- |
| Professor Frank Buttgereit | Professor Ingvar Bjarnason | Professor Luigi Biasucci | Carl Davis |
| **Rheumatologists** | Dr Xavier Calvet | Professor José González-Juanatey | Dr John Fabule |
| Dr Adewale Adebajo | Professor Stanislas Chaussade | Professor Olivier Gurné | Nicholas Lagan |
| Professor Olavi Airaksinen | Dr Antonio Curado | Dr Matthias Hermann | Natacha Oukli |
| Professor Garcia Llorente | Professor Wolfgang Fischbach | Professor James Ritter | Dr Nathalie ter Wengel |
| Professor Ernest Choy | Dr Jean-Louis Frossard | **Clinical Pharmacologists** | Dr Chris Walker |
| Professor Augusto Faustino | Dr. Mario Guslandi | Professor Corrado Blandizzi |  |
| Professor Markus Gaubitz | Professor Ulf Haglund | Professor Carmelo Scarpignato |  |
| Professor Lennart Jacobsson | Professor Richard Hunt | **Evidence-based Scientists** |  |
| Professor Herbert Kellner | Professor Ernst Kuipers | Dr Andrew Moore |  |
| Professor Willem Lems | Professor Angel Lanas |  |  |
| Dr Mike Nurmohamed | Professor Peter Malfertheiner |  | **No-Voting Observers: Lucid** |
| Professor Alfonso Oriente | Professor Hubert Piessevaux |  | Julie Borland |
| Professor Mart van de Laar |  |  | Cherry Bwalya |
|  |  |  | Emma Cox |
